# In Vivo Release of Vancomycin from Calcium Phosphate Cement

**DOI:** 10.1155/2018/4560647

**Published:** 2018-05-15

**Authors:** Kentaro Uchida, Ken Sugo, Takehiko Nakajima, Mitsufumi Nakawaki, Shotaro Takano, Naoshige Nagura, Masashi Takaso, Ken Urabe

**Affiliations:** ^1^Department of Orthopedic Surgery, Kitasato University School of Medicine, 1-15-1 Kitasato, Minami, Sagamihara, Kanagawa 252-0375, Japan; ^2^Research and Development Department, HOYA Technosurgical Corporation, 1-1-110 Tsutsujigaoka, Akishima, Tokyo 196-0012, Japan; ^3^Department of Orthopedic Surgery, Kitasato University Medical Center, 6-100 Arai, Kitamoto, Saitama 364-8501, Japan

## Abstract

Calcium phosphate cement (CPC) has good release efficiency and has therefore been used as a drug delivery system for postoperative infection. The release profile of CPC has mainly been evaluated by* in vitro* studies, which are carried out by immersing test specimens in a relatively large amount of solvent. However, it remains unclear whether antibiotic-impregnated CPC has sufficient clinical effects and release* in vivo*. We examined the* in vivo* release profile of CPC impregnated with vancomycin (VCM) and compared this with that of polymethylmethacrylate (PMMA) cement. To evaluate the release profile* in vitro*, the test specimens were immersed in 10 mL sterile phosphate-buffered saline per gram of test specimen and incubated at 37°C for 56 days in triplicate. For* in vivo* experiments, the test specimens were implanted between the fascia and muscle of the femur of rats. Residual VCM was extracted from the removed test specimens to determine the amount of VCM released into rat tissues. CPC released more VCM over a longer duration than PMMA* in vitro*. Released levels of VCM from CPC/VCM* in vivo* were 3.4-fold, 5.0-fold, and 8.6-fold greater on days 1, 7, and 28, respectively, than those released on the corresponding days from PMMA/VCM and were drastically greater on day 56 due to inefficient release from PMMA/VCM. The amount of VCM released from CPC and PMMA was much higher than the minimum inhibitory concentration (1.56 *μ*g) and lower than the detection limit, respectively. Our findings suggest that CPC is a suitable material for releasing antibiotics for local action against established postoperative infection.

## 1. Introduction

Periprosthetic joint infection after total joint arthroplasty is a serious complication that requires prompt treatment. The two-stage exchange procedure is an effective treatment option for such infections [1]. Intravenously administered antibiotics, such as vancomycin (VCM), have poor tissue transferability depending on the site of infection, which decreases their therapeutic potential [2]. The therapeutic potential of VCM can be improved by combining it with a carrier to increase its retention at infection sites in the first stage of the procedure [3].

Calcium phosphate cement (CPC) has good release efficiency and has therefore been used as a drug delivery system for postoperative infection. CPC can release a greater volume of antibiotic over a longer duration than polymethylmethacrylate (PMMA) cement [4–7], a finding that has also been confirmed clinically [8–10]. Antibiotic release from the cement material is triggered when external solvent penetrates the material's pores, causing the antibiotic to diffuse out of the material [11, 12].* In vitro* studies are carried out by immersing test specimens in a relatively large amount of solvent, thereby providing ideal conditions for antibiotic release. However, it is unclear whether such release also occurs* in vivo* because tissue fluid is expected to only fill a few implanted sites compared to* in vitro* studies in the field of orthopedic surgery.

Here, we implanted CPC impregnated with VCM into rat tissue between the fascia and muscle and evaluated the release profile* in vivo*. We also compared the release profiles* in vitro*.

## 2. Materials and Methods

### 2.1. Materials

CPC (Biopex-R Advance) and PMMA (Surgical Simplex P Bone Cement) were obtained from HOYA Technosurgical (Tokyo, Japan) and Stryker (Tokyo, Japan), respectively. Injectable VCM hydrochloride was purchased from Shionogi (Osaka, Japan). All other chemicals were obtained from Wako Pure Chemical Industries (Osaka, Japan).

### 2.2. Preparation of Test Specimens

**Figure 1:**
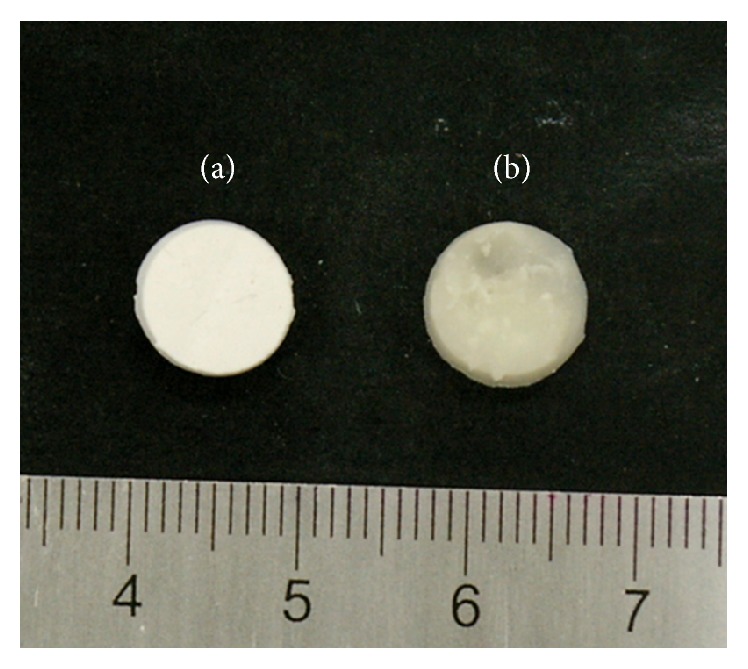
Photo of test specimens prepared at *φ* 10 mm ×* t* 2 mm. Calcium phosphate cement/vancomycin (CPC/VCM) (a) and polymethylmethacrylate (PMMA)/VCM (b).

All preparations were performed aseptically. CPC powder (24 g), VCM (2 g), and a dedicated solvent (5.6 mL) were uniformly mixed to make a paste before adding a further 1.6 mL of solvent for effective handling (final volume 7.2 mL). The amount of VCM used (2 g) was the same as that used by our clinical team. The paste was applied to a silicone sheet containing 60 molds (*φ* 10 mm ×* t* 2 mm) and was hardened by incubating for 3 h at room temperature. For PMMA, polymer powder (24 g), VCM (2 g), and liquid monomer (12 mL) were uniformly mixed to make a paste and hardened in the same manner as for CPC. Once hardened, the molds were removed to obtain the test specimens, which were designated CPC/VCM and PMMA/VCM (Figure 1). The 60 total CPC/VCM and PMMA/VCM specimens were used as follows: 50 for* in vivo* testing, 3 for* in vitro* testing, and the remaining for morphological observation.

### 2.3. In Vitro Study

The test specimens were immersed in 10 mL sterile phosphate-buffered saline (PBS(-)) per gram of test specimen and incubated at 37°C for 56 days in triplicate. PBS(-) was replaced daily. Eluates were collected on days 1, 7, 28, and 56 (*n* = 3 each). VCM was detected by high-performance liquid chromatography (HPLC) on the day of collection.

### 2.4. Determination of VCM Concentration

A series of standard solutions of differing known concentrations of VCM in PBS(-) were prepared and injected into a CAPCELL-PAK C18 UG120 column (5 *μ*m, *φ* 4.6 mm ×* h* 250 mm; Shiseido, Tokyo, Japan) of an Elite LaChrom HPLC system (Hitachi High-Technologies, Tokyo, Japan) equipped with a L-2455 Diode Array Detector (Hitachi High-Technologies). The HPLC conditions were as follows: column temperature, 30°C; mobile phase A, triethylamine buffer (pH 3.2)/acetonitrile/tetrahydrofuran = 92/7/1 (v/v); isocratic elution, phase A (20 min); flow rate, 1 mL/min; wavelength, 280 nm; and injection volume, 20 *μ*L. The peak area of VCM in each standard solution was measured and plotted against the VCM concentration to generate a calibration curve. The concentration of VCM in each eluate sample was then determined by HPLC using the same conditions as those used to generate the standard calibration curve.

### 2.5. In Vivo Study

*In vivo *studies were approved by the Kitasato University School of Medicine and Hospital Ethics Committee (Approval number 2017-098). Wistar rats, 10 weeks old, were anesthetized with 0.3 mg/kg medetomidine (Domitor, Nippon Zenyaku Kogyo, Fukushima, Japan), 0.5 mg/kg butorphanol (Vetorphale, Meiji Seika Pharma, Tokyo, Japan), and 0.5 mg/kg midazolam (Midazolam Sandoz, Sandoz, Tokyo, Japan). CPC/VCM and PMMA/VCM were implanted between the fascia and the muscle of the femur on the left and right side of 40 rats, respectively. The fascia and skin were sutured and the animals were allowed to move freely in their cages immediately after the surgery. Test specimens were removed on days 1, 7, 28, and 56, after sacrificing the animals (*n* = 10 each).

### 2.6. Extraction of VCM

Residual VCM was extracted from the removed test specimens on days 1, 7, 28, and 56 to determine the amount of VCM released into rat tissues. CPC/VCM was removed and ground in a mortar, and 10 mg of the powder was completely dissolved in 0.3 mL of 1 M hydrochloric acid, followed by 2.7 mL of water. The solution was subsequently diluted 10-fold with chromatographic mobile phase solution. PMMA/VCM was removed and crushed using an osteotome, and 10 mg pieces were completely dissolved in 3 mL of acetone. The solution was sonicated for 1 min (UR-20P, Tomy Seiko, Tokyo, Japan), diluted 10-fold with chromatographic mobile phase solution, and centrifuged at 5,000 ×g for 5 min at 25°C (3700, Kubota, Tokyo, Japan) to remove precipitates. Ten of each test specimen without implantation were used as a reference with 0% release. All extracted VCM was analyzed using HPLC as described above.

### 2.7. Morphological Observation

The relationship between the pore structure and VCM release profiles of CPC/VCM and PMMA/VCM was analyzed and compared. CPC/VCM without implantation was immersed in 20 mL of acetone for 10 min for dehydration, removed, and dried at room temperature; this process was not necessary for PMMA/VCM. Thin sections (1-2 mm thickness) of each test specimen were prepared using a microtome for scanning electron microscopy (SEM) analysis (S-4300, Hitachi High-Technologies) and the analysis of pore size distribution using mercury porosimetry (AutoPore IV9520, Micromeritics, Norcross, GA, USA).

### 2.8. Statistical Analysis

The VCM release profile and the total amount of VCM released by CPC/VCM and PMMA/VCM were statistically compared using a* t*-test following a Levene test for equality of variance result of *p* ≥ 0.05. Differences were considered significant if *p* values were less than 0.05.

## 3. Results

### 3.1. In Vitro Study

**Figure 2:**
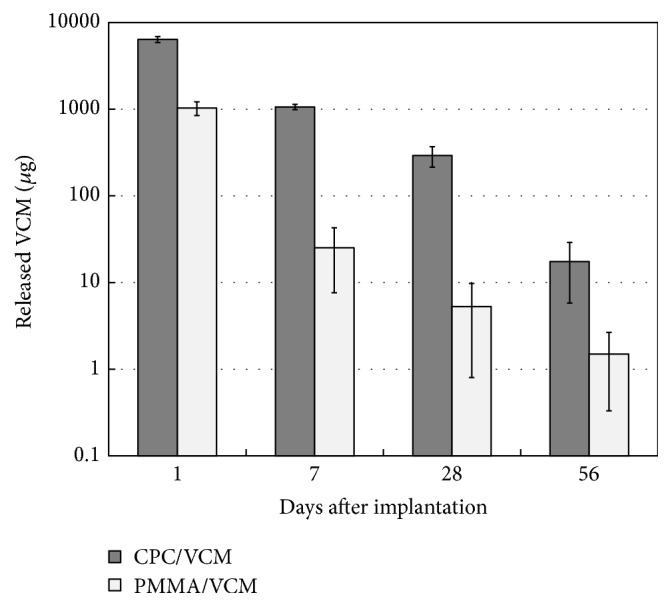
*In vitro* release of VCM from CPC/VCM and PMMA/VCM per day. Solvent was replaced daily and VCM levels were examined on days 1, 7, 28, and 56 by high-performance liquid chromatography. The average value and standard deviation in triplicate are shown.

The amount of VCM released from CPC/VCM was greater than that from PMMA/VCM throughout the test period (Figure 2).

### 3.2. In Vivo Study

**Figure 3 fig3:**
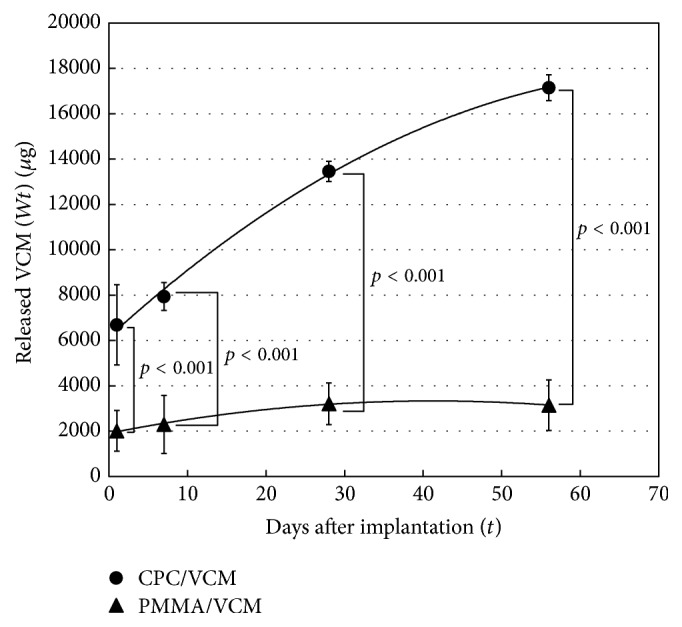
*In vivo* accumulated release of VCM from CPC/VCM and PMMA/VCM. Test specimens were removed from rats on days 1, 7, 28, and 56, and the amount of VCM released into rat tissues was examined. Horizontal axis “*t*” and vertical axis “*W*_*t*_” indicate the number of days after implantation and the amount of VCM released from each test specimen, respectively. The average value and standard deviation of 10 of each specimen are shown as *W*_1_, *W*_7_, *W*_28_, and *W*_56_.

The amount of VCM released into rat tissues (*W*_*t*_) was calculated by subtracting the amount of VCM extracted from the removed test specimens from that extracted from the reference specimens without implantation. The symbol *t* (= 1, 7, 28, and 56) indicates the number of days after implantation.  Figure 3 shows *W*_1_, *W*_7_, *W*_28_, and *W*_56_ for CPC/VCM and PMMA/VCM, which represent the average amount of VCM released from 10 specimens removed on each day. CPC/VCM released significantly more VCM over a longer duration than PMMA* in vivo,* as was observed* in vitro* (*p* < 0.001, for all measured time points).

**Figure 4 fig4:**
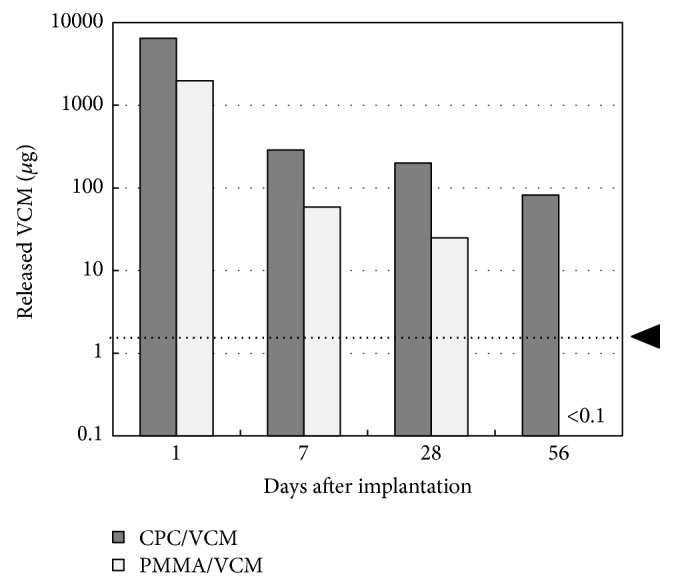
*In vivo* release of VCM from CPC/VCM and PMMA/VCM per day. “*W*_*t*_-*W*_*t*−1_” values were calculated from the approximate curve in Figure 3. The closed arrowhead indicates the minimum inhibitory concentration (1.56 *μ*g).

Similar to the* in vitro* studies described above, we examined the amount of VCM released per day instead of accumulated release. We generated an approximate curve from *W*_1_, *W*_7_, *W*_28_, and *W*_56_ (Figure 3) and calculated “*W*_*t*_-*W*_*t*−1_” using the curve to determine the amount of VCM released per day. The amount of VCM released on days 1, 7, 28, and 56 is shown in Figure 4. In contrast to *W*_1_, *W*_7_, *W*_28_, and *W*_56_ values, which were calculated as the average of 10 data points, *W*_*t*−1_ was calculated from the approximate curve as one data point; there are therefore no error bars for data shown in Figure 4. The release pattern of VCM from CPC/VCM into rat tissues was similar to that observed in the* in vitro* study. The mean amount of VCM released on day 56 (79.8 *μ*g) was much higher than the minimum inhibitory concentration (MIC) of 1.56 *μ*g per milliliter of local body fluid [13] for methicillin-resistant* Staphylococcus aureus*. While the release pattern of VCM from PMMA/VCM was also similar to that observed* in vitro*, the mean amount of VCM released on day 56 (<0.1 *μ*g) was lower than the detection limit and the MIC. Released levels of VCM from CPC/VCM were 3.4-fold, 5.0-fold, and 8.6-fold greater on days 1, 7, and 28, respectively, than those released on the corresponding days from PMMA/VCM and were drastically greater on day 56 due to inefficient release from PMMA/VCM.

### 3.3. Comparison of Total Amount Released

**Figure 5:**
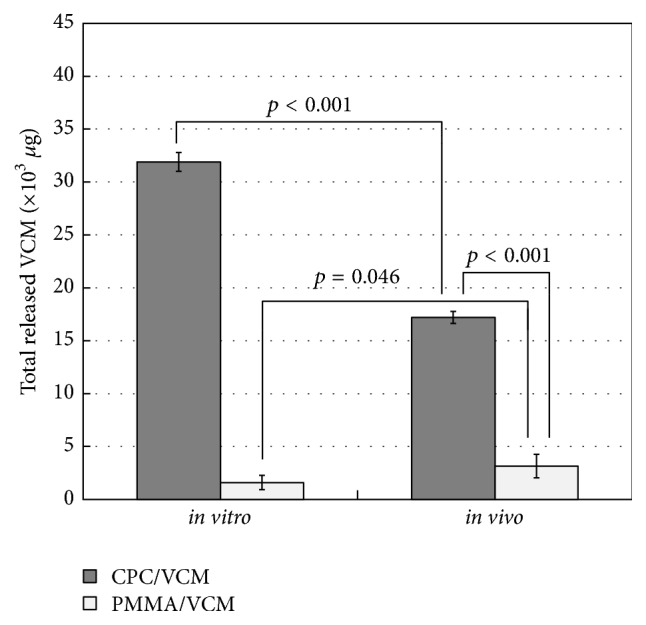
Total amount of VCM released from CPC/VCM and PMMA/VCM into rat tissues in 56 days.* In vivo* data for day 56 are directly cited from Figure 3 (*W*_56_).* In vitro* data were calculated by summing values from the elution curves generated following the daily exchange of solvent. The difference between* in vitro* and* in vivo* values indicates that CPC/VCM has the potential to continue to release residual VCM after day 56* in vivo*. The average value and standard deviation of 10* (in vivo)* and 3* (in vitro)* of each specimen are shown.

Figure 5 shows the total amount of VCM released from CPC/VCM and PMMA/VCM into rat tissues in 56 days. The values for the* in vivo* study are indicated by the data on day 56 in Figure 3 (*W*_56_) and those for the* in vitro* study were calculated by summing values from the elution curves generated following the daily exchange of solvent. The total amount of VCM released by CPC/VCM* in vivo* was approximately half that released* in vitro*, at elution rates of 64.1% and 119.3% (*p* < 0.001), respectively. Almost all impregnated VCM was released from CPC/VCM* in vitro*, and our approximate curve suggests that CPC/VCM has the potential to continue to release residual VCM fractions after day 56* in vivo*. In contrast, the total amount of VCM released by PMMA/VCM* in vivo *was 2-fold greater than that released* in vitro*, at elution rates of 29.6% and 14.8% (*p* = 0.046), respectively. The total amount of VCM released* in vivo* from CPC/VCM was 5.5-fold greater than that released from PMMA/VCM (*p* < 0.001).

### 3.4. SEM Observation and Porosimetry Analysis

**Figure 6:**
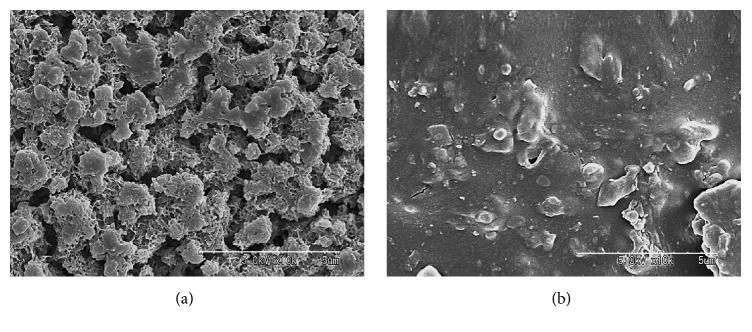
SEM images of the surfaces of CPC/VCM (a) and PMMA/VCM (b). Scale bars: 5 *μ*m.

**Figure 7:**
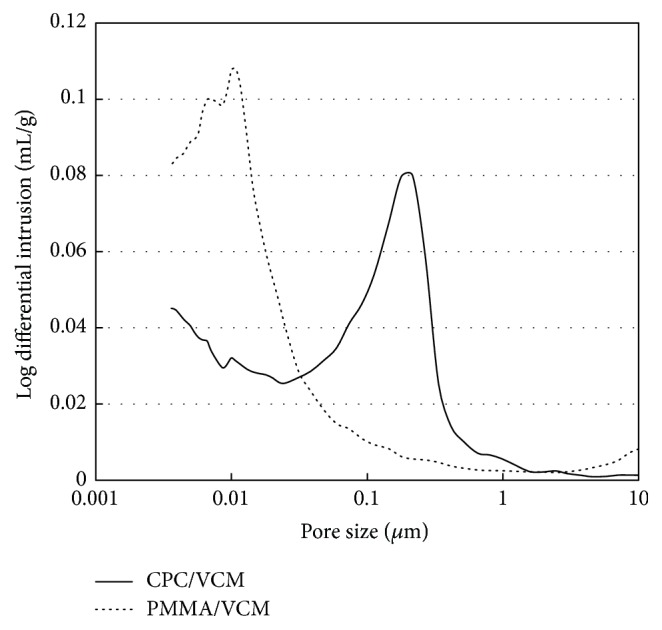
Mercury porosimetry analysis for CPC/VCM and PMMA/VCM. Mercury porosimetry plot of the pore sizes in CPC/VCM and PMMA/VCM. CPC/VCM and PMMA/VCM had pore sizes in the range of 0.025–0.65 *μ*m and 0.0036–0.074 *μ*m, respectively.

SEM observation showed numerous pores on the surface of CPC/VCM (Figure 6(a)), which are formed by crystal growth and entanglement of *α*-tricalcium phosphate particles [14]. In contrast, little to no pores were observed on the surface of PMMA/VCM (Figure 6(b)), which is formed by radical polymerization and is fundamentally different from the composition of CPC/VCM. Consistent with the SEM analysis, mercury porosimetry revealed that the test specimens had markedly different pore size distributions, with mode values of 0.22 *μ*m and 0.011 *μ*m for CPC/VCM and PMMA/VCM, respectively (Figure 7).

## 4. Discussion

Previous studies have reported that antibiotic-loaded bone cement spacers are useful for antibiotic release and inhibiting bacterial growth [7, 15, 16]. PMMA cement has long been proposed to permit release of impregnated antibiotics* in vitro* and* in vivo* [16, 17] and is still used as a standard antibacterial spacer, especially in mechanical strength-required sites [7]. The poor release of antibiotics from PMMA compared to CPC* in vitro* has been reported [4–7]. However, to date, the release profile of these materials has not been determined* in vivo*. Here, PMMA/VCM showed 2-fold greater release of VCM* in vivo* compared to* in vitro* (Figure 5). This suggests that PMMA/VCM may be partly phagocytosed by macrophages as a foreign body* in vivo* [18], which would cause the elution rate to increase, despite there being less fluid volume around the material. However, VCM release from PMMA/VCM* in vivo* almost completely stopped after day 28 (Figure 3). In the two-stage exchange procedure following infected total joint replacement, reconstruction surgery is generally performed 6–8 weeks (or 12 weeks or more depending on the case) after the initial debridement and implantation of the antibiotic-impregnated cement material [19–21]. Therefore, according to our findings, PMMA/VCM is unable to provide antibiotic release for the clinically required period, despite showing longer release duration than that reported* in vitro*.

The ability of CPC to maintain high antibiotic concentrations in a relatively large amount of solvent has mainly been confirmed by* in vitro* studies [4–7]. Additionally, however, there are many published reports on the treatment of periprosthetic infections using antibiotic-impregnated CPC as a potential alternative to conventional PMMA cement in clinical settings [8–10]. In the present study, CPC/VCM released more VCM over a longer duration than PMMA/VCM* in vivo* and* in vitro* (Figures 2 [Fig fig3] [Fig fig4]–5). The amount of VCM released* in vivo* was approximately half that released* in vitro*, which may be due to less fluid volume around the material in the former compared to latter condition (Figure 5). Additionally, VCM release from CPC/VCM* in vivo* may continue after day 56 (Figure 3), with the approximate curve predicting release for the next 18 days (total 74 days). Given that the amount released per day exceeds the MIC (1.56 *μ*g) for more than 10 weeks after the initial implantation, our calculations suggest that CPC/VCM may provide sufficient antibacterial effect for the clinically required period. Therefore, CPC/VCM, which can provide antibiotic release for more than 8 weeks, may be a suitable material for providing local antibiotics to combat established postoperative infections.

Reports have indicated that an antibiotic release profile depends on the pore size of the cement material [12, 22]. The pore size of CPC impregnated with VCM and gentamicin is smaller than that for CPC impregnated with VCM alone, with the former showing decreased antibiotic elution compared to the latter [12]. In the present study, CPC/VCM had 20-fold larger pore size compared to PMMA/VCM (Figures 6 and 7). Therefore, the larger pore size of CPC/VCM may underlie its good release profile* in vivo*.

We observed long term release of VCM from CPC/VCM* in vivo*. However, long term use of CPC/VCM may be associated with problems such as resistance among staphylococci, including vancomycin-intermediate* Staphylococcus aureus* (VISA) [23], heteroresistant vancomycin-intermediate* Staphylococcus aureus* strains (hVISA) [24], and vancomycin-resistant* Staphylococcus aureus* (VRSA) [25], and cement stability in clinical use. Further investigations are needed to determine the efficacy and safety of this treatment for established postoperative infections.

## 5. Conclusions

CPC/VCM showed longer retention time than PMMA/VCM* in vitro* and* in vivo*. The properties of CPC/VCM suggest that it is a promising agent for the treatment of established postoperative infections in clinical settings.

## Data Availability

All data generated or analyzed during this study are included in this published article.
